# Social Media Coverage of Scientific Articles Immediately After Publication Predicts Subsequent Citations - #SoME_Impact Score: Observational Analysis

**DOI:** 10.2196/12288

**Published:** 2020-04-17

**Authors:** Niranjan Jude Sathianathen, Robert Lane III, Declan G Murphy, Stacy Loeb, Caitlin Bakker, Alastair D Lamb, Christopher J Weight

**Affiliations:** 1 Department of Urology University of Minnesota Minneapolis, MN United States; 2 Department of Surgical Oncology Peter MacCallum Cancer Centre Melbourne Australia; 3 Department of Urology New York University New York, NY United States; 4 Department of Population Health New York University New York, NY United States; 5 Health Science Libraries University of Minnnesota Minneapolis, MN United States; 6 Nuffield Department of Surgical Sciences University of Oxford Oxford United Kingdom; 7 Department of Urology Churchill Hospital Cancer Centre Oxford University Hospitals NHS Foundation Trust Oxford United Kingdom

**Keywords:** bibliometrics, online social networking, online systems, online intervention, social media

## Abstract

**Background:**

Social media coverage is increasingly used to spread the message of scientific publications. Traditionally, the scientific impact of an article is measured by the number of citations. At a journal level, this conventionally matures over a 2-year period, and it is challenging to gauge impact around the time of publication.

**Objective:**

We, therefore, aimed to assess whether Web-based attention is associated with citations and to develop a predictive model that assigns relative importance to different elements of social media coverage: the #SoME_Impact score.

**Methods:**

We included all original articles published in 2015 in a selection of the highest impact journals: The New England Journal of Medicine, The Lancet, the Journal of the American Medical Association, Nature, Cell, and Science. We first characterized the change in Altmetric score over time by taking a single month’s sample of recently published articles from the same journals and gathered Altmetric data daily from the time of publication to create a mixed effects spline model. We then obtained the overall weighted Altmetric score for all articles from 2015, the unweighted data for each Altmetric component, and the 2-year citation count from Scopus for each of these articles from 2016 to 2017. We created a stepwise multivariable linear regression model to develop a #SoME_Score that was predictive of 2-year citations. The score was validated using a dataset of articles from the same journals published in 2016.

**Results:**

In our unselected sample of 145 recently published articles, social media coverage appeared to plateau approximately 14 days after publication. A total of 3150 articles with a median citation count of 16 (IQR 5-33) and Altmetric score of 72 (IQR 28-169) were included for analysis. On multivariable regression, compared with articles in the lowest quantile of #SoME_Score, articles in the second, third, and upper quantiles had 0.81, 15.20, and 87.67 more citations, respectively. On the validation dataset, #SoME_Score model outperformed the Altmetric score (adjusted R^2^ 0.19 vs 0.09; *P*<.001). Articles in the upper quantile of #SoME_Score were more than 5 times more likely to be among the upper quantile of those cites (odds ratio 5.61, 95% CI 4.70-6.73).

**Conclusions:**

Social media attention predicts citations and could be used as an early surrogate measure of scientific impact. Owing to the cross-sectional study design, we cannot determine whether correlation relates to causation.

## Introduction

A direct reflection of the digital age, scientific work is primarily disseminated Web-based rather than in the conventional hard copy format [1]. The academic community has embraced the internet as a medium for discussion and debate with the increasing emergence of social media–facilitated journal clubs [2]. Although these facets of academia have evolved with the times and technology, measures of scientific impact still generally rely on the traditional citation counts and lag behind. Furthermore, there are important limitations to using citation count in this manner [3], primarily the time required for citations to mature, and therefore, impactful articles can only be classified as so retrospectively when they may already be obsolete. It is increasingly clear that the digital footprint harbored by each item of Web-based information contains a wealth of data that can be used to make inferences about it. Altmetric is a platform that captures the Web-based attention received by academic articles from several sources, including news, blogs, Twitter, Facebook, Sina Weibo, Wikipedia, policy documents, Q&A, F1000/Publons/PubPeer, YouTube, Reddit/Pinterest, LinkedIn, Open Syllabus, Google+, and Patents. It includes a wealth of information that could be used to circumvent the time delay to formal citations and thereby provide an earlier measure of scientific impact. This has substantial implications on a communal and individual level. These Web-based attention metrics can be used to potentially identify scientific works that are *game changers* prospectively, which would increase awareness of these works and potentially lead to earlier implementation of the recommendations arising from their results. Similarly, individuals will be able to receive credit for their work years earlier, which may form the foundation for further work and success because they could conceivably leverage this early indicator of scientific merit to endorse their applications for sponsorship or support [4].

Initial work in this area has demonstrated that Web-based metrics are associated with scholarly impact. Eysenbach [5] reported that the top-tweeted articles from the Journal of Medical Internet Research (highly tweeted after 7 days compared to other articles in the same issue) are also more likely to be the most highly cited articles from that journal. This relationship has also been confirmed in ecological journals [6]. Thelwall et al [7] also demonstrated a moderate correlation between Twitter, Facebook, research highlights, blogs, mainstream media, forums, Q&A, and citations in a heterogeneous sample of articles. However, there was no overall weighted score at the time of research because the Altmetric system was still undergoing development and has evolved considerably since then, with different components now being included. In addition, these works have mostly focused on articles from a single journal, and therefore, the applicability of these findings to other journals is unclear. It should also be noted that Web-based academic presence has grown tremendously since 2013, and thus, these results may not be applicable today. Nonetheless, there is a growing body of evidence that Web-based attention metrics are associated with scholarly impact and urge the scientific community to recognize this and attribute credit accordingly. This is further reflected by the increasing Web-based presence of journals that further potentiate the dissemination of research [8].

We aimed to characterize the dynamics in Altmetric scores over time immediately following publication and assess whether there is an association between Altmetric score or any of its components and citation count for articles published in high-impact scientific and clinical journals. We further intended to develop a new index that can be used to predict scientific impact soon after publication.

## Methods

### Sample

All articles published in Cell, Nature, Science, the Journal of the American Medical Association, the New England Journal of Medicine, and The Lancet during the 2015 calendar year were included for analysis. We used articles from the 2016 calendar year from the same journals as a test set. The latter 3 journals were considered to be clinically oriented journals, whereas the former 3 were classified as being scientifically oriented. We selected these journals because they represent high-impact publications in the clinical and scientific fields. Information on each of these publications was obtained from Scopus by matching digital object identifiers (DOIs), including the type of document and yearly cumulative citation counts. Only publications classified as *Articles* or *Articles in Press* in Scopus were included in the analysis to only assess original articles; that is, publications classified as *Editorial*, *Conference Paper*, *Letter*, *News*, *Note*, *Review*, and *Short Survey* were excluded because they were not believed to be peer-reviewed, novel scientific contributions. We also excluded articles with missing classifications. We obtained Altmetric data for each of the included publications by matching DOIs. This included the automatically calculated, weighted Altmetric score generated by Altmetric, which is an approximation of the attention a particular research output has received based on the raw number of news, blogs, Twitter, Facebook, Sina Weibo, Wikipedia, policy documents, Q&A, F1000/Publons/PubPeer, YouTube, Reddit/Pinterest, LinkedIn, Open Syllabus, Google+, and Patents. We also obtained the individual weightings for each of the elements in calculating the Altmetric score that are available Web-based. We included the impact factor of each journal as a continuous covariable in our model.

Altmetric counts are cumulative and cannot be obtained for retrospective periods, and therefore, the data represent the total Altmetric score at the time of search (October 2017). Therefore, to characterize the change in Altmetric score and establish that the total Altmetric score at the time of search represents the meaningful Altmetric score early after article publication, we included articles from the same journal that was published within 2 days of the Altmetric search and tracked their Altmetric score daily for the first fortnight and then every 3 days for the next fortnight and then weekly for the last 2 weeks.

### Statistical Analysis

Before analysis, normal distribution and homogeneity of variances were assessed. Data that were not normally distributed were described using median and IQR and compared using the Kruskal-Wallis test. The correlation between 2-year citation counts and Altmetric scores—weighted and individual components—was estimated by calculating the Spearman rank correlation coefficient, as the dependent variables were not normally distributed. To develop a #SoME_Score that could be used to predict citations, we performed a multivariable linear model using the forward stepwise regression method based on Akaike information criteria to determine the best fitting model [9]. We adjusted for each component of the Altmetric score in addition to time since publication. We defined outliers as those having a Cook distance 4 times greater than the mean.

To assess the change in Altmetric score from the time of publication, we fit mixed effects spline models. Cubic spline models were chosen because the data were nonlinear, and therefore, the data were allowed to fit separate curves for each section of time. Cubic spline knots were placed at 5-day intervals to characterize the change in score over this time frame.

All *P* values were 2 sided, and the statistical significance set at the .05 level. Data analysis was performed in R (R Foundation for Statistical Computing) version 3.4.

## Results

### Inclusion Criteria

A total of 3510 articles met the inclusion criteria and were included for analysis. Of these, 15.52% (545/3510), 28.34% (995/3510), 25.25% (883/3510), 8.43% (296/3510), 11.22% (394/3510), and 11.31% (397/3510) were published in Cell, Nature, Science, the Journal of the American Medical Association, the New England Journal of Medicine, and The Lancet, respectively. Included articles had a median citation count of 37 (IQR 12-74). There was a difference in median citations between articles in clinical and scientific journals (32 vs 39; *P*<.001).

**Figure 1:**
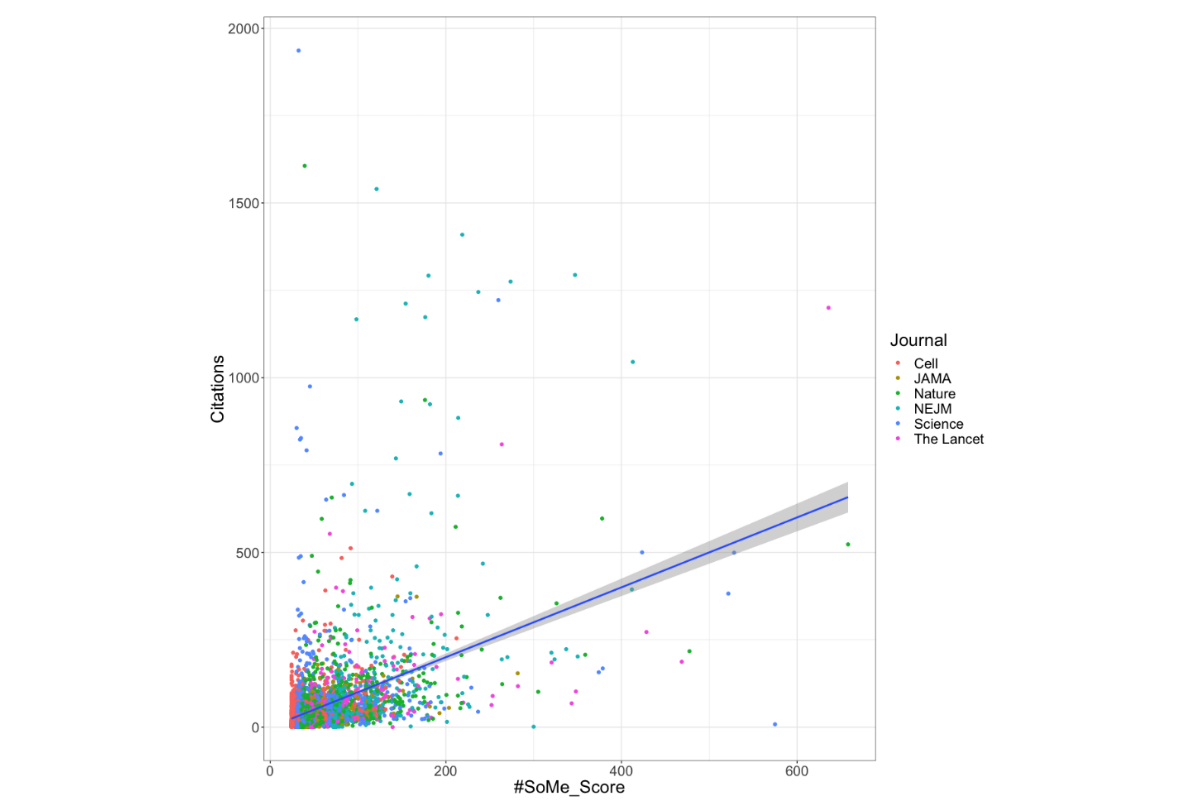
Association between #SoME_Score and citation count. JAMA: Journal of the American Medical Association; NEJM: New England Journal of Medicine.

The median Altmetric score for all the articles was 72 (IQR 28-169). There was no difference in the Altmetric score between clinical and scientific journals (72 vs 72; *P*=.10). The adjusted R^2^ of the Altmetric model in the training set was 0.045. With stepwise regression, only news, blog, policy, Peer Review, Wiki, F1000, and the journal impact factor were included in the model to generate the #SoME_Score (Multimedia Appendix 1). The median #SoME_Score for all articles was 47.86 (IQR 36.73-76.58). On multivariable regression, the #SoME_Score had a significant impact on citation count (Figure 1; *P*<.001). The adjusted R^2^ of the #SoME_Score model in the training set was 0.168, which was significantly better than the Altmetric model (*P*<.001). Compared with articles in the lowest quantile of #SoME_Score, articles in the second, third, and upper quantiles had 0.81 (SE 5.5), 15.20 (SE 5.5), and 87.67 (SE 5.5) more citations, respectively. Articles in the upper quantile of #SoME_Score were more than 5 times more likely to be among the upper quantile of those cites (odds ratio [OR] 5.61, 95% CI 4.70-6.73). Conversely, publications in the top quantile of the Altmetric score did not act as a predictor of high citation count (OR 0.98, 95% CI 0.34-2.88).

On the validation dataset, #SoME_Score model outperformed the Altmetric score (adjusted R^2^ 0.19 vs 0.09; *P*<.001).

### Altmetric Score Trend

**Figure 2:**
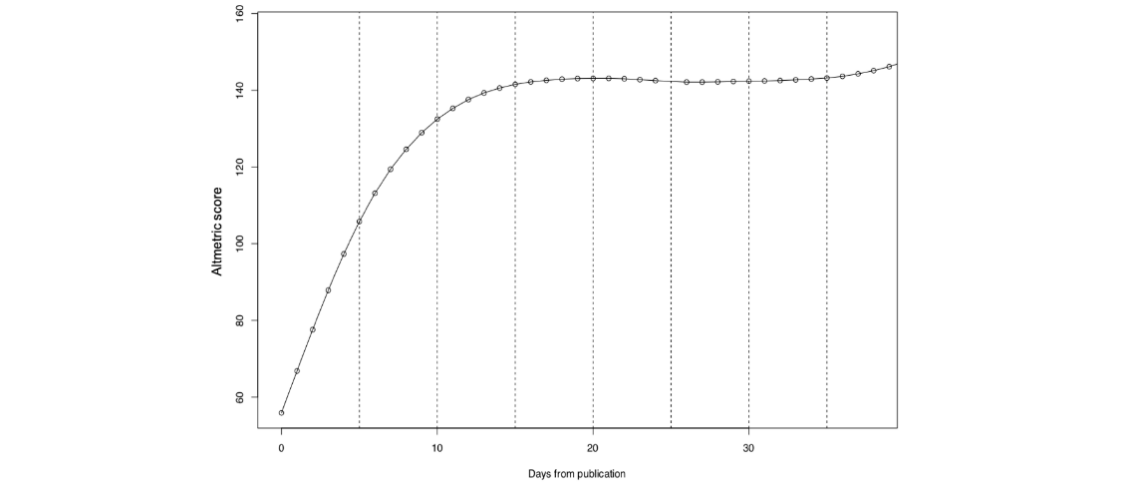
Altmetric score trend.

A total of 145 articles published in the same 6 journals between September 18 and September 22, 2017, were included in the analysis for the Altmetric score trend. Data were collected on the cumulative Altmetric score of these articles for a mean 40 days after publication. The increase in the Altmetric score appears to plateau approximately 14 days after publication (Figure 2).

### Journal of Medical Internet Research Validation Results

We evaluated the use of the #SoME_Score in articles published in the Journal of Medical Internet Research in the 2016 calendar year. Of the 420 articles included in this analysis, the median Altmetric score was 8 (IQR 4-19), and the median 2-year citation count was 6 (IQR 3-11). #SoME_Score acted as a significant predictor of citations in this external validation dataset (*P*<.01). Articles that were among the highest quartile of #SoME_Scores were nearly 3 times more likely to be among the highest quartile of those cited (OR 2.88, 95% CI 1.71-4.83). In contrast to the primary dataset of articles, articles in the Journal of Medical Internet Research in the top 25% of Altmetric scores were also more likely to be those with a high citation count (OR 3.28, 95% CI 2.02-5.30).

## Discussion

### Principal Findings

We observed an association between social media attention of scholarly articles and academic impact measured by citation count. Furthermore, we were able to identify the most important components of the Altmetric score that are associated with citations and then developed a new index, the #SoME_Score, which can be calculated in the immediate postpublication period (2-3 weeks) and be used to predict scholarly impact years down the road. We suggest that this score may be best used to predict high-impact papers as defined as those with citations in the highest quartile. This is in line with the findings from the study by Eysenbach [5], who reported that highly tweeted articles (measured by the Twimpact Factor, which is the number of tweets after 7 days) were 11 times more likely to be highly cited than less tweeted articles. However, the Twimpact factor was only generated from articles published in the Journal of Medical Internet Research, which inherently are likely to generate more Web-based attention because of the nature of its subject. Our findings were shown to be robust across other medical and scientific journals, which often report on topics that are not easily discerned by the general public. It should be noted that these findings represent an association only and not causation, that is, high Web-based presence may lead to increased citations or the impactful nature of certain studies may be underpinning the early Web-based attention it receives [5].

It is important to note that not all components of the Altmetric score were correlated with citations, which, therefore, served as the impetus for the development of the #SoME_Score. Only the news, blog, policy, Peer Review, and F1000 components showed a meaningful correlation to citation count. The overall Altmetric score is derived by applying weights to each component, which correlates to its *attention*, that is, its exposure and engagement in the Web-based sphere. This is a clear shortcoming of using the overall Altmetric score to predict academic impact because the weights applied are not a reflection of the contribution of each component to citation count. Aside from the limited sample, this could partly explain previous studies, which did not find a relationship between Altmetric and citations [10]. Furthermore, the overall Altmetric score is not specific for the scientific community as the measure of *attention* used to determine the weights will be largely driven by the general population of which academics only form a small proportion. This is why articles on populous subjects, such as sex and politics, have phenomenally high Altmetric scores but relatively low citation counts (Multimedia Appendices 2 and 3). In contrast, the weights applied to each component in the #SoME_Score is specific because citations are driven by academics. These fundamental benefits of the #SoME_Score suggest that it could be adopted as a predictive index of scholarly impact.

To the best of our knowledge, this is the first study to evaluate the trend in Altmetric scores for scientific articles and show an early plateau within 2 weeks of publication. This highlights both the speed of information dissemination in the internet age and the short window of spotlight a publication receives. Furthermore, the fleeting nature of Web-based attention is partly a reflection of the sheer volume of scientific work entering the literature [11]. The number of articles entering PubMed annually has increased by 62.5% between 2003 and 2013 [12]. We are, therefore, faced with the challenge of filtering signal from the noise [13]. #SoME_Impact Score could help amplify the signal.

### Limitations

In terms of limitations, we only included articles from high-impact clinical and scientific journals to improve our power to detect a relationship, given the increased average citations of these publications. Given that these journals are highly selective for impact stories, our findings may not be generalizable to works in other journals, and the #SoME_Impact Score would need further validation to confirm similar predictive ability. However, we have reported on the generalizability of this score to articles published in the Journal of Medical Internet Research. Furthermore, the Web-based presence of biomedical academics is continually evolving with, for example, increasing numbers engaging in Twitter, and therefore, it is possible that components that were shown not to have a correlation with citation count will become significant in the future [14]. Moreover, we only evaluated total and individual components included in the Altmetric score, and further research could explore whether there are alternative metrics or components that can be used to improve predictive ability.

### Conclusions

Social media attention can be used as an early surrogate measure of academic impact. This could lead to academics being recognized and receiving early credit for their work instead of having to wait for citation counts to mature. Further work is required to validate these findings in the wider biomedical literature and nonbiomedical fields.

## Appendix

Multimedia Appendix 1 Beta coefficients for #SoME_Score.

Multimedia Appendix 2 Outliers.

Multimedia Appendix 3 Association between #SoMe_Score and citation count with outliers.

## Abbreviations

DOI: digital object identifier
OR: odds ratio
