# Supplementary material for: Social Media Coverage of Scientific Articles Immediately After Publication Predicts Subsequent Citations - #SoME_Impact Score: Observational Analysis
Source: J Med Internet Res. 2020 Apr 17;22(4):e12288. doi: 10.2196/12288 (PMC7195668; doi:10.2196/12288)
Supplement: Multimedia Appendix 1 [file jmir_v22i4e12288_app1.docx]

**Appendix 1. Beta coefficients for #SoME_Score**

| **Component** | **Coefficient** | **Standard Error** | **P value** |
| --- | --- | --- | --- |
| F1000 | 35.67 | 6.51 | <0.001 |
| Policy | 15.37 | 2.36 | <0.001 |
| Wiki | 3.69 | 0.55 | <0.001 |
| Peer Review | 0.94 | 0.40 | 0.018 |
| Blog | 0.95 | 0.39 | 0.015 |
| News | 0.64 | 0.12 | <0.001 |
| Impact factor | 1.00 | 0.13 | <0.001 |
