# Supplementary material for: Social Media Coverage of Scientific Articles Immediately After Publication Predicts Subsequent Citations - #SoME_Impact Score: Observational Analysis
Source: J Med Internet Res. 2020 Apr 17;22(4):e12288. doi: 10.2196/12288 (PMC7195668; doi:10.2196/12288)
Supplement: Multimedia Appendix 2 [file jmir_v22i4e12288_app2.docx]

**Appendix 2. Outliers**

| Title | Journal | Altmetric | Citations |
| --- | --- | --- | --- |
| Smart food policies for obesity prevention | The Lancet | 173 | 117 |
| Nivolumab versus Docetaxel in Advanced Squamous-Cell Non-Small-Cell Lung Cancer. | NEJM | 378 | 1540 |
| Combined Nivolumab and Ipilimumab or Monotherapy in Untreated Melanoma. | NEJM | 370 | 1409 |
| PD-1 Blockade in Tumors with Mismatch-Repair Deficiency. | NEJM | 749 | 1294 |
| Possible artifacts of data biases in the recent global surface warming hiatus | Science | 2349 | 168 |
| Ezetimibe Added to Statin Therapy after Acute Coronary Syndromes. | NEJM | 525 | 885 |
| A new antibiotic kills pathogens without detectable resistance | Nature | 2899 | 523 |
| The geographical distribution of fossil fuels unused when limiting global warming to 2¬†¬∞C. | Nature | 2485 | 217 |
| Contribution of job control and other risk factors to social variations in coronary heart disease incidence. | The Lancet | 72 | 68 |
| Ibrutinib as Initial Therapy for Patients with Chronic Lymphocytic Leukemia. | NEJM | 932 | 202 |
| A Randomized Trial of Intensive versus Standard Blood-Pressure Control. | NEJM | 1151 | 1045 |
| Kew, O. et al. Outbreak of poliomyelitis in Hispaniola associated with circulating type 1 vaccine-derived poliovirus. Science 296, 356-359 | Science | 50 | 44 |
| Randomized Assessment of Rapid Endovascular Treatment of Ischemic Stroke. | NEJM | 379 | 1245 |
| Endovascular Therapy for Ischemic Stroke with Perfusion-Imaging Selection. | NEJM | 266 | 1212 |
| Mutational landscape determines sensitivity to PD-1 blockade in non‚Äìsmall cell lung cancer | Science | 242 | 1222 |
| Tissue-based map of the human proteome | Science | 435 | 783 |
| Will Ebola change the game? Ten essential reforms before the next pandemic. The report of the Harvard-LSHTM Independent Panel on the Global Response to Ebola | The Lancet | 1186 | 102 |
| Effectiveness of a hospital-wide programme to improve compliance with hand hygiene | The Lancet | 77 | 187 |
| Plastic waste inputs from land into the ocean | Science | 2782 | 382 |
| Stent-Retriever Thrombectomy after Intravenous t-PA vs. t-PA Alone in Stroke. | NEJM | 260 | 932 |
| Pembrolizumab versus Ipilimumab in Advanced Melanoma. | NEJM | 221 | 1173 |
| Pembrolizumab for the Treatment of Non-Small-Cell Lung Cancer. | NEJM | 121 | 1167 |
| Human Chromosome 7: DNA Sequence and Biology | Science | 23 | 8 |
| High-performance photovoltaic perovskite layers fabricated through intramolecular exchange | Science | 53 | 1936 |
| Global, regional, and national incidence, prevalence, and years lived with disability for 301 acute and chronic diseases and injuries in 188 countries, 1990‚Äì2013: a systematic analysis for the Global Burden of Disease Study 2013 | The Lancet | 2148 | 1200 |
| Comparison of ixekizumab with etanercept or placebo in moderate-to-severe psoriasis (UNCOVER-2 and UNCOVER-3): results from two phase 3 randomised trials | The Lancet | 490 | 185 |
| Glypican-1 identifies cancer exosomes and detects early pancreatic cancer. | Nature | 380 | 354 |
| Effect of intensive blood-glucose control with metformin on complications in overweight patients with type 2 diabetes (UKPDS 34). UK Prospective Diabetes Study (UKPDS) Group. | The Lancet | 86 | 809 |
| Estimating the reproducibility of psychological science | Science | 3300 | 157 |
| Empagliflozin, Cardiovascular Outcomes, and Mortality in Type 2 Diabetes. | NEJM | 729 | 1275 |
| Nivolumab versus Everolimus in Advanced Renal-Cell Carcinoma. | NEJM | 515 | 924 |
| Nivolumab versus Docetaxel in Advanced Nonsquamous Non-Small-Cell Lung Cancer. | NEJM | 405 | 1292 |
