# Supplementary material for: Social Media Coverage of Scientific Articles Immediately After Publication Predicts Subsequent Citations - #SoME_Impact Score: Observational Analysis
Source: J Med Internet Res. 2020 Apr 17;22(4):e12288. doi: 10.2196/12288 (PMC7195668; doi:10.2196/12288)
Supplement: Multimedia Appendix 3 [file jmir_v22i4e12288_app3.docx]

**Appendix 3. Association between #SoMe_Score and citation count with outliers**
